# The Therapeutic Potential of Aprepitant in Glioblastoma Cancer Cells through Redox Modification

**DOI:** 10.1155/2022/8540403

**Published:** 2022-03-03

**Authors:** Soodabeh Rezaei, Reza Assaran Darban, Hossein Javid, Seyed Isaac Hashemy

**Affiliations:** ^1^Department of Biology, Faculty of Sciences, Mashhad Branch, Islamic Azad University, Mashhad, Iran; ^2^Department of Clinical Biochemistry, Faculty of Medicine, Mashhad University of Medical Sciences, Mashhad, Iran; ^3^Department of Medical Laboratory Sciences, Varastegan Institute for Medical Sciences, Mashhad, Iran; ^4^Surgical Oncology Research Center, Mashhad University of Medical Sciences, Mashhad, Iran

## Abstract

Although there is no doubt regarding the involvement of oxidative stress in the development of glioblastoma, many questions remained unanswered about signaling cascades that regulate the redox status. Given the importance of the substance P (SP)/neurokinin 1 receptor (NK1R) system in different cancers, it was of particular interest to evaluate whether the stimulation of this cascade in glioblastoma-derived U87 cells is associated with the induction of oxidative stress. Our results showed that SP-mediated activation of NK1R not only increased the intracellular levels of malondialdehyde (MDA) and reactive oxygen species (ROS) but also reduced the concentration of thiol in U87 cells. We also found that upon SP addition, there was a significant reduction in the cells' total antioxidant capacity (TAC), revealing that the SP/NK1R axis may be involved in the regulation of oxidative stress in glioblastoma cells. The significant role of SP/NK1R in triggering oxidative stress in glioblastoma has become more evident when we found that the abrogation of the axis using aprepitant reduced cell survival, probably through exerting antioxidant effects. The results showed that both MDA and ROS concentrations were significantly reduced in the presence of aprepitant, and the number of antioxidant components of the redox system increased. Overall, these findings suggest that aprepitant might exert its anticancer effect on U87 cells through shifting the balance of oxidant and antioxidant components of the redox system.

## 1. Introduction

As one of the most challenging malignancies to treat, glioblastoma has an adverse prognosis and poor quality of life [1]. Numerous therapeutic interventions have been developed for managing this cancer; however, these were not successful enough to induce a complete remission [2]. Recurrence of the tumor is also inevitable in glioblastoma, which makes the development of more accurate and less toxic treatment strategies more crucial [3].

Numerous molecular investigations have introduced oxidative stress as a hallmark of the progression of many cancers, including glioblastoma [4–6]. Despite the importance of oxidative stress in the pathogenesis of glioblastoma, the precise molecular mechanism responsible for the regulation of this event has not yet been identified. Recently, attention has been attracted to tachykinins, as they have a critical movement in the pathogenesis of glioblastoma [7] and control both oxidative stress and antioxidant systems [8]. Substance P (SP) is a small neuropeptide that binds to the tachykinins receptor 1 (TACR1), also known as neurokinin 1 receptor (NK1R), which is one of the most important G-protein-coupled tachykinin receptors initially found in central and peripheral nervous systems [9]. Within a short time, NK1R expression was found in other tissues, and the discovery of noncanonical activities of SP/NK1R added a new perspective to this signaling axis as a regulator of tumorigenesis [7, 10].

In addition to its numerous biological functions, the SP/NK1R signaling pathway plays a significant role in tumor formation due to its ability to regulate cell proliferation and sustains cancer cell survival [11, 12]. The ability to regulate oxidative stress has brought attention to the SP/NK1R axis, especially in glioblastoma, as it can provide an opportunity for cancer cells to increase their metabolic activity. Baek et al. indicated that SP induced cell damage in retinal pigmented cells through PI3K-mediated induction of reactive oxygen species (ROS) production [13]. This finding has opened a new window into the role of the SP/NK1R axis in glioblastoma pathogenesis and provided an opportunity to use the NK1R antagonist for the treatment of this malignancy. Thus far, the anticancer property of several NK1R antagonists has been tested in different glioblastoma-derived cell lines and xenograft models; however, efforts are still underway to find a drug that has maximal tumor suppressor activity and minimal side effects.

Among different synthesized NK1R antagonists, aprepitant is a competing nonpeptide antagonist of the NK1R, first incorporated into a moderately emetogenic chemotherapy regimen to prevent chemotherapy-induced nausea and vomiting [14, 15]. When it was demonstrated that aprepitant could conveniently pass the blood-brain barrier and block the center of nausea in the brain through halting the attachment of substance P (SP) to NK1R [16], it has been assumed that this agent might have antitumor properties. In xenograft models, aprepitant reversed SP-induced mitogenic stimulation and reduced tumor burden [17]. The abrogation of NK1R using aprepitant in leukemic cell lines was also associated with the induction of G1 cell cycle arrest and caspase-3-dependent apoptotic cell death [18]. Moreover, Berger et al. demonstrated that aprepitant reduced the proliferative capacity of hepatoblastoma *in vivo* and *in vitro* investigations [19]. Apart from monotherapy, there are also several studies suggesting that aprepitant may be a good candidate as an adjunctive drug alongside chemotherapy [20]. The results of a completed phase III trial also shed light on a favorable pharmacokinetic and safety profile for aprepitant [21]. Although multiple studies emphasized the antitumor effect of aprepitant in several cancers, still, there is little evidence on the precise mechanism of action of this NK1R antagonist in human cancers.

Given these, in the present study, we aimed to investigate whether there is a correlation between the activation of the SP/NK1R axis and the induction of oxidative stress in glioblastoma-derived U87 cells. Moreover, it was of particular interest to evaluate whether blockage of this signaling using aprepitant could reduce the viability of the cells via changing the balance of the redox system in favor of antioxidant property in U87 cells.

## 2. Materials and Methods

### 2.1. Cell Lines and Reagents

Glioblastoma-derived U87 cells were cultured in DMEM medium supplemented with antibiotics, 10% fetal bovine serum, and 2 mmol/L L-glutamine (Invitrogen) in the presence of 5% CO_2_ at 37°C. For evaluating the effect of NK1R stimulation, cells were treated with substance P (SP) at the concentrations of 400 and 800 nM. A stock solution of aprepitant (Santa Cruz Biotechnology Inc., Dallas, USA) was provided by dissolving the agent in sterile dimethyl sulfoxide (DMSO) for the drug treatment. As a negative control, we added an equal volume of DMSO to the drug/SP-untreated cells.

### 2.2. MTT Assay

To evaluate whether the treatment of U87 cells with aprepitant is coupled with the reduction of cell survival, cells were treated with increasing concentrations of the agent, and the metabolic activity of the cells was examined after 24 h using microculture tetrazolium assay. We plated 8000 U87 cells in each well of 96-well plate, and then, the cells were incubated with indicated concentrations of aprepitant 0 (control), 9.3, 18.7, 37.5, 75, 150, and 200 *μ*M. After 24 h, the media was discarded, and the cells were further incubated with MTT solution (5 mg/mL in PBS) at 37°C for 3 h. Afterward, we dissolved the resulting formazan with DMSO, and the absorption was evaluated at 570 nm in an enzyme-linked immunosorbent assay reader.

### 2.3. Malondialdehyde (MDA) Assay

To investigate the concentration of MDA upon exposure to SP (400 and 800 nM) in the absence or presence of aprepitant (20 *μ*M), we used malondialdehyde (MDA) assay kit (Kushan Zist, Tehran, Iran). To prepare the cells, 1.2 × 10^6^ U87 cells were lysed in 1X BHT buffer. After centrifugation at 14000 g for 5 min, 250 *μ*L of samples was added to 500 *μ*L TCA, and the mixture was incubated for 5 min at 95°C. For precipitation of the proteins, the mixture was centrifuged at 14000 g for 5 min. Then, 500 *μ*L of the supernatant was added to 250 *μ*L of TBA buffer, and the mixture was incubated at 95°C for 30 min. The absorption of samples was then measured at 532 nm. To evaluate MDA concentration in U87 cells, we plotted a standard curve from the obtained results using GraphPad Prism software.

### 2.4. ROS Assay

To investigate whether the activation of the SP/NK1R axis in U87 cells is coupled with ROS production, the cells were cultured in a 96-well plate in the presence of SP, aprepitant, or the combination of two agents. After the indicated time interval, the media was discarded from the plate, and 100 *μ*L of Ready assay buffer from cellular reactive oxygen species (ROS) assay kit (Kushan Zist, Tehran, Iran) was added to each well. After removing the Ready buffer, 100 *μ*L of DCF staining buffer was added to each well, except the one considering the blank well. The plate was then incubated at 37°C for 60 min and incubated with 100 *μ*L of R3 stimulator for an additional 20 min in the dark. After discarding the solution and DCF staining buffer, the cells were washed twice with Ready assay buffer and the production of ROS was evaluated by measuring the fluorescence intensity at a wavelength between 480-500 nm. The final concentration of ROS was measured by using a standard curve.

### 2.5. TAC Assay

U87 cells were treated with SP (400 nM and 800 nM), aprepitant (20 *μ*M), or in the combined modality. After the indicated time, 1 × 10^6^ cells were harvested for evaluating the total antioxidant capacity (TAC) using the total antioxidant capacity (TAC) assay kit (Kushan Zist, Tehran, Iran). The harvested cells were lysed by repeated cycles of freezing and thawing and then centrifuged at 12000 g for 15 min. The supernatant was collected and transferred into the fresh tube. Afterward, 150 *μ*L, 10 *μ*L, and 30 *μ*L from R2, R3, and %5 reading buffers were added to the samples, respectively. After 5 min of incubation at room temperature, the absorbance of the samples was evaluated at 734 nm using a plate reader. The final concentration of TAC was measured using a standard curve.

### 2.6. Thiol Assay

The level of total sulfhydryl groups was assessed by the DTNB (2,2′-dinitro-5,5′-dithiodibenzoic acid) reduction method. This reagent reacts with SH groups to produce a yellow color (peak absorbance 412 nm). Briefly, 0.1 mL of Tris–EDTA buffer (pH 8.6) was exposed to the 0.05 mL cell lysate, and after that, the absorbance was measured at 412 nm against Tris–EDTA buffer alone (A1). Next, 20 *μ*L DTNB reagent (10 mM in methanol) was added to the mixture, and after 15 min of incubation at room temperature, the sample absorbance was reread (A2). Additionally, the absorbance of the DTNB reagent alone was measured as a blank group (B). Finally, total SH concentration (*μ*M) was obtained by the following equation: total thiol concentration (*μ*M) = (A2 − A1 − B) × 1.07/0.05 × 13.6.

### 2.7. Statistical Analysis

The results are presented as the mean ± standard deviation of three independent experiments. All the investigations were performed in triplicate to provide a meaningful result. The significance of the differences between experimental variables, a probability level of *P* < 0.05, was demonstrated using two statistical tests, two-tailed Student's *t*-test, and one-way variance analysis. The data analysis was performed by using SPSS and GraphPad prism software.

## 3. Results

### 3.1. The Antitumor Effect of Aprepitant on the Survival Rate of U87 Cells

**Figure 1:**
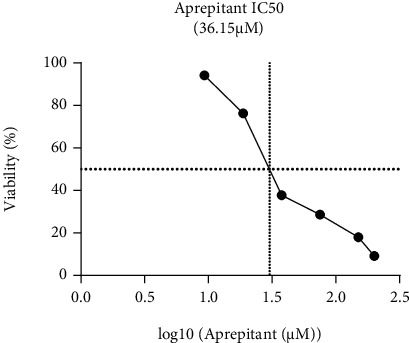
The anticancer effect of aprepitant on glioblastoma cells. The results of the MTT assay showed that aprepitant could remarkably reduce the viability of U87 cells with an estimated IC50 value of 36.15 *μ*M.

Previous studies have declared that exposure of malignant cells to aprepitant is coupled with the reduction in the survival and proliferative capacity of the cells. Given these, it was of particular interest to evaluate the antitumor activity of this NK1R antagonist in glioblastoma-derived U87 cells. We treated the cells with the increasing concentrations of aprepitant, and then, the drug-treated cells were subjected to MTT assay. As presented in Figure 1, we found that upon blockage of the NK1R signaling axis using aprepitant, there was a significant reduction in the viability of the cells. Our results showed that aprepitant at the concentration of 20 *μ*M effectively diminished the number of U87 viable cells. Moreover, the estimated IC50 value for aprepitant in U87 cells was 36.15 *μ*M. These findings were suggestive of the antitumor activity of aprepitant in glioblastoma-derived cells.

### 3.2. Evaluating the Impact of the SP/NK1R Signaling Axis on MDA Level

**Figure 2:**
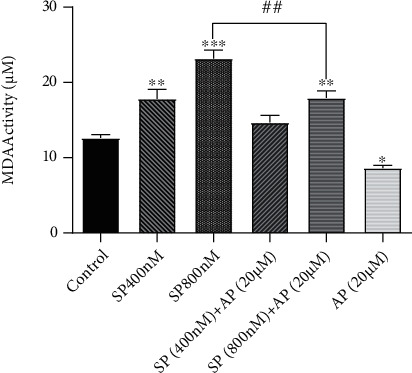
The effect of NK1R stimulation and suppression on the concentration of MDA in U87 cells. While SP increased the concentration of MDA in U87 cells, the treatment of glioblastoma cells using aprepitant (20 *μ*M) diminished the level of this oxidative marker in the cells. Moreover, our results showed that aprepitant could attenuate the effect of SP on the production of MDA. Values are given as the mean ± S.D. of three independent experiments. ## is representative of *P* ≤ 0.05, which is statistically significant.

The interconnection between oxidative stress and tumorigenesis has been well-established in several reports. It has been claimed that cancer cells exploit the redox system to maintain their survival and proliferative capacity. We aimed to evaluate whether the activation of the NK1R signaling axis in glioblastoma cells could augment the oxidative stress in the cells. We treated U87 cells with SP, a well-known ligand of NK1R, and then, we evaluated the amount of malondialdehyde (MDA), a well-known marker of lipid peroxidation. As depicted in Figure 2, we found that upon SP addition to the culture media of U87 cells, there was a concentration-dependent increase in the amount of MDA. It was demonstrated that SP at the concentration of 800 nM could elevate the concentration of MDA approximately to 25 *μ*M, suggestive of the potent role of the SP/NK1R system in disrupting the balance of the redox system.

Moreover, to confirm that the elevated MDA concentration was due to the stimulation of the SP/NK1R axis in the U87 cells, we treated the cells with the antagonist of NK1R. Of note, our results showed that aprepitant (20 *μ*M), as a single agent, could reduce the concentration of MDA in U87 cells (Figure 2). The favorable activity of aprepitant against oxidative stress became more evident, when we simultaneously treated the cells with aprepitant and sp. As presented in Figure 2, there was a significant reduction in the concentrations of MDA upon exposure of the cells to SP-plus-aperient, suggestive of the antioxidant property of aprepitant in the malignant cells.

### 3.3. The NK1R Stimulation using SP Increased the Amount of ROS in U87 Cells

**Figure 3:**
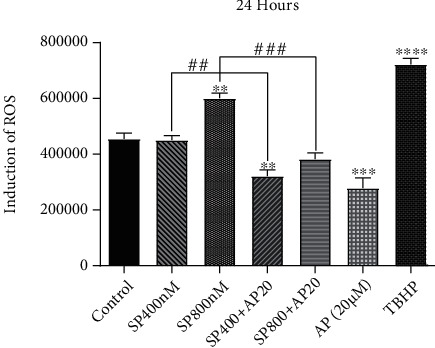
The effect of NK1R on the production of ROS in glioblastoma-derived cells. Using the ROS assay, we evaluated the intracellular level of ROS after the treatment of U87 cells with SP, aprepitant, or the combination of both agents. SP significantly elevated the intracellular level of ROS in U87 cells at the concentration of 800 nM. Aprepitant, either as a single agent or in combination with SP, could halt the generation of free radicals in malignant cells. Values are given as the mean ± S.D. of three independent experiments. ## and ### are representative of *P* ≤ 0.05, which is statistically significant.

To investigate whether NK1R activation in SP-treated U87 cells was associated with the induction of oxidative stress, the intracellular amount of reactive oxygen species (ROS) was evaluated by the ROS assay. In agreement with the results of the MDA assay, treatment of the cells with SP at the concentration of 800 nM significantly elevated the intracellular level of ROS (Figure 3), indicating that SP-mediated stimulation of the NK1R resulted in the induction of oxidative stress probably through increasing the production of ROS in the cells. Interestingly, when this signaling axis was suppressed by aprepitant, either alone or in the presence of SP, there was a robust reduction in the total production of ROS (Figure 3). As presented, aprepitant, as a single agent, decreased the oxidative stress in U87 cells, but this agent also prevented the SP-induced ROS production in the cells.

### 3.4. The Oxidative Effect of Activated the SP/NK1R in U87 Cells Was Coupled with the Reduction in Thiol Group Concentration

**Figure 4:**
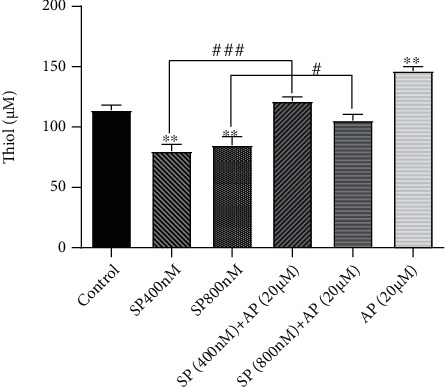
Stimulation of NK1R using SP decreased the concentration of the thiol component in U87 cells. Using the thiol assay, the concentration of proteins containing the thiol group was evaluated in U87 cells after either SP or aprepitant treatment. Unlike SP, which decreased the level of thiol in the cells, aprepitant increased the concentration of thiol-containing proteins, suggestive of the antioxidant property of this agent. Values are given as the mean ± S.D. of three independent experiments. # and ### are representative of *P* ≤ 0.05, which is statistically significant.

Given the effect of activated NK1R in the induction of oxidative stress in U87 cells, we aimed to investigate whether SP-mediated stimulation of the NK1R could reduce the activity of the antioxidant system. The thiol group is a well-known component of many antioxidant enzymes used by the redox system to compensate for the harmful effects of free radical spices. The results of the thiol assay revealed that when SP was added to the culture medium of U87 cells, there was a significant reduction in the concentration of the thiol groups. To gain insights into the antioxidant effects of aprepitant in U87 cells, we scrutinized the thiol group concentration upon exposure of the cells to this agent. As evident in Figure 4, we found that culturing the cells with 20 *μ*M concentration of aprepitant resulted in a marked elevation in thiol group concentration. Additionally, the analysis of the antioxidant property of U87 cells treated with aprepitant in combination with SP showed an accumulation in the number of thiol groups. As presented in Figure 4, the addition of aprepitant to SP-treated cells increased thiol group concentrations considerably.

### 3.5. Activation of the SP/NK1R Signaling Axis Decreased the Total Antioxidant Capacity (TAC) of U87 Cells

**Figure 5:**
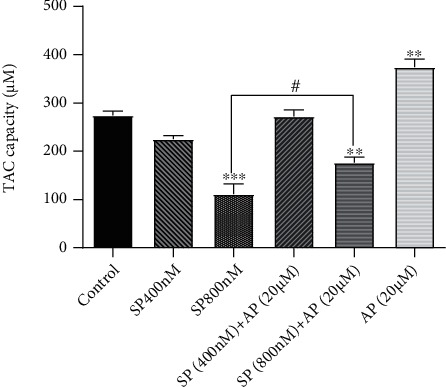
Stimulation of NK1R using SP is coupled with the reduction in the total antioxidant capacity of U87 cells. When U87 cells were treated with SP at the concentration of 800 nM, there was a significant reduction in the concentration of TAC. However, blockage of NK1R using aprepitant (20 *μ*M) resulted in an approximately 2-fold increase in the concentration of TAC in U87 cells. Moreover, in the presence of aprepitant, the ability of SP to reduce the concentration of TAC significantly decreased. Values are given as the mean ± S.D. of three independent experiments. # is representative of *P* ≤ 0.05, which is statistically significant.

Having established that SP-mediated activation of the NK1R signaling axis in U87 cells was coupled with the reduction in the antioxidant property of the cells, it was of particular interest to evaluate the effect of this network on the total antioxidant capacity (TAC) of U87 cells. We found that SP significantly reduced the TAC of U87 cells with the maximal repression observed in the presence of SP at the concentration of 800 nM (Figure 5). Additionally, the ablation of the NK1R using aprepitant (20 *μ*M) was coupled with the remarkable elevation in the TAC capacity, indicating a correlation between the activated NK1R and the reduction of antioxidant capacity of the malignant cells. Previous studies have shown that the induction of the antioxidant process in cancer cells is a promising approach to prevent the rate of tumorigenesis. Accordingly, when we exposed SP-treated cells to aprepitant, we found that this agent could potently compensate the oxidative property of SP, which was in agreement with the results obtained from the thiol group assay. As presented in Figure 5 and compared with SP-treated cells, aprepitant at the concentration of 20 *μ*M was successful in preventing the repressive effect of SP (800 nM) on the TAC capacity U87 cells. While the TAC capacity of U87 cells reached 100 *μ*M in the presence of SP (800 nM), the addition of aprepitant to the culture media increased this capacity up to 200 *μ*M (Figure 3). Taken together, these findings suggested that while SP/NK1R stimulation in U87 cells was associated with the elevation of oxidative stress, blockage of this cascading using aprepitant exerted anticancer effects via altering the balance of the redox system in favor of antioxidant properties.

## 4. Discussion

Despite striking attempts to ameliorate cancer treatment strategies and improve patient outcome, efforts have not successfully reached the desired results due to the engagement of multiple factors, such as activation of oxidative stress. Recent disclosures showed that changes in redox state in tumorigenesis may correlate with tolerance to chemotherapeutic drugs [22]. A new perspective has been aroused in the treatment strategy that proposes adding antioxidant agents may be befitting for therapeutic protocols. This approach seems to be promising. Nevertheless, since the role of the precise signaling axis in the activation of oxidative stress is not well-established, finding a new agent to change the balance of the redox system in favor of antioxidant property has been postponed.

The importance of the SP/NK1R in the pathogenesis of human cancers coupled with its association with the acquisition of chemoresistance phenotype has raised the question that perhaps the involvement of this axis in these events is mediated through regulation of oxidative stress [7]. Although several studies have examined the effect of aberrantly activated AP/NK1R signaling pathways on the pathogenies of the different human cancers, the connection between this axis and the induction of oxidative stress has not yet been clarified. The results of the present study showed that upon the SP-mediated NK1R activation, there was a remarkable elevation in the concentration of malondialdehyde (MDA) and reactive oxygen species (ROS) in glioblastoma cells; a human malignancy in which the induction of oxidative stress is coupled not only with disease progression but also with poor prognosis [23, 24].

Several lines of evidence have declared that MDA, which is the well-known marker of lipid peroxidation and oxidative stress, could promote the risk of cancer development, including breast cancer [25] and ovarian carcinoma [26]. Lipid peroxidation is also notorious for its key role in inducing chemoresistance against temozolomide (TZM) in glioblastoma cells [27]. It is demonstrated that upon lipid peroxidation and MDA formation, the intracellular amount of ROS increases in neoplastic cells, reducing the cells' sensitivity to chemotherapeutic drugs via activating DNA damage responses [28]. ROS production in neoplastic cells; however, it is a matter of debate. There is a wealth of evidence suggesting that excessive ROS generation is coupled with the induction of apoptotic cell death [29]. It is shown that inhibiting the NK1R in acute myeloid leukemia (AML) cells increases the intracellular levels of ROS and, in turn, induces mitochondria-mediated apoptotic cell death [30]. In acute lymphoblastic leukemia (ALL), it has been claimed that through elevating the intracellular levels of ROS, aprepitant could reduce the expression of antiapoptotic proteins [18]. Many chemotherapeutic drugs such as doxorubicin, cisplatin, and etoposide also eliminate the population of neoplastic cells by requiring ROS [31]. Despite the advantages, the excessive production of ROS within the cancer cells is not always beneficial, as this reactive oxygen species could protect cancer cells from the anticancer agents by increasing the expression of multidrug-resistant proteins (MRDs), overcoming cell cycle arrest, and altering the activity of autophagy flux [32]. The variant of ROS could also facilitate tumor cell migration and metastasis and attenuate the cytotoxicity of the chemotherapeutic drugs [33]. The harmful effect of ROS and its related oxidative stress on the activity of thiol-containing proteins, which have a critical function in maintaining the balance of the redox system and mediating the reversible posttranslational modifications [34], could also be other reasons suggesting that these free radicals might act in favor of tumorigenesis.

In agreement with these findings and the significant elevation of ROS, we found that the activation of the NK1R in U87 cells was coupled with the decrease in the intracellular level of thiol content. This finding suggested that perhaps the results of the constant stimulation of the NK1R using SP in glioblastoma cells are ROS production, which enhances the carcinogenesis process by disturbing the regulation of the redox system. The oxidative property of the SP/NK1R also became more evident when aprepitant, a well-known blocker of the SP receptor, decreased the intracellular level of MDA and ROS in U87 cells and increased the concentration of thiol in the malignant cells. Moreover, we found that the antioxidant property of aprepitant was also coupled with the reduction of the survival of the cells, as revealed by the significant decrease in the metabolic activity of U87 cells. This finding was in accordance with the results of the previous investigations, which reported the cytotoxic property for aprepitant in several cancers ranging from solid tumors [35] to hematologic malignancies [36]. Ghahremanloo et al. also suggested that a single agent of aprepitant diminishes the viability of colon cancer-derived SW480 cells by reducing the intracellular levels of ROS and abrogating the NF-*κ*B signaling axis [37]. In glioblastoma, Korfi et al. suggested that a higher concentration of aprepitant (35 *μ*M) is capable of reducing ROS production while increasing the enzymatic activity of superoxidase dismutase (SOD) and catalase [38]. Aprepitant also showed antioxidant activity in glioblastoma-derived cell lines by reducing the expression of thioredoxin reductase [39]. It has also been reported that this NK1R antagonist suppresses superoxide activity in glioblastoma-induced rats by inhibiting neutrophil activity [40]. To the best of our knowledge, this was the first time that the antioxidant activity of the lower concentrations of this agent (20 *μ*M) has been tied with its anticancer effects, and our study suggested that aprepitant probably reduced the survival of glioblastoma cells via blocking the oxidative stress.

Different factors have been evaluated to determine patients' response rates to both conventional and novel therapeutic approaches in the modern era of cancer management. For a long time, antioxidant components have been claimed to reduce the risk of cancer [22]. However, when epidemiological studies indicated that there is a correlation between the serum level of antioxidant components and the response rate to chemotherapeutic drugs, the common perspective of the antioxidant compound has changed and total antioxidant capacity (TAC) has been introduced as a promising marker for evaluating the prognosis of human cancers. In a study conducted by Santiago-Arteche et al., it was shown that the TAC level was significantly lower in metastatic colorectal cancer patients as compared to patients without metastasis [41]. Likewise, several studies have demonstrated that the serum level of TAC is remarkably lower in breast cancer patients than in healthy counterparts [42, 43]. Although the reduction of the serum level of TAC is reported in patients with glioblastoma [24], its precise molecular mechanism has not been elucidated.

**Figure 6:**
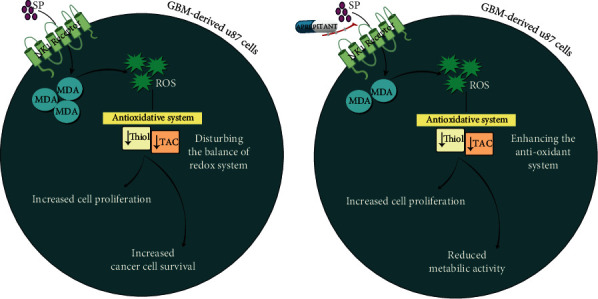
Schematic presentation. Once SP binds to NK1R, the intracellular levels of MDA and ROS increase in glioblastoma-derived U87 cells, which in turn through disturbing the antioxidant system reinforce the survival and the proliferative capacity of neoplastic cells. Aprepitant, an antagonist of NK1R, on the other hand, prevents the oncogenic activity of SP. This agent abrogates the activity of MDA and subsequently the production of ROS within the malignant cells. Moreover, through potentiating the activity of the antioxidant system, as revealed by the elevation in the activity of thiol and TAC, aprepitant decreases the metabolic activity of U98 cells.

Accordingly, as shown in Figure 6, the present study results showed that the stimulation of the NK1R signaling pathway in glioblastoma-derived U87 cells could be a probable mechanism leading to the reduction of TAC concentration. Moreover, our results showed that aprepitant could effectively bypass the suppressive effect of SP on TAC activity. We found that in the presence of aprepitant, there was a significant elevation in TAC concentrations in U87 cells. The data presented in this study, on the one hand, suggested the aberrant activated SP/NK1R signaling axis as a probable mediator of induction of oxidative stress in glioblastoma cells and, on the other hand, showed that aprepitant could exert anticancer effect on U87 cells by shifting the balance between oxidant and antioxidant components of the redox system. However, since the results presented in this study are obtained based on one cell line and glioblastoma is notorious for its heterogeneous nature [44], further analysis on other glioblastoma cell lines with different genetic characterization is required to more precisely study the role of the SP/NK1R axis in tumorigenesis. Moreover, it should be well-established whether other abnormalities, such as hyperactivation of EGFR, could reinforce the stimulation of NK1R and thereby attenuate the therapeutic value of aprepitant.

## Data Availability

Data are available upon request.
